# Guest editorial: Caring in the 21st century: research evidence and knowledge generation

**DOI:** 10.1111/hsc.12169

**Published:** 2014-10-28

**Authors:** Mary Larkin, Alisoun Milne

**Affiliations:** 1 Faculty of Health and Social Care, The Open UniversityMilton Keynes, UK; 2 School of Social Policy, Sociology and Social Research, University of KentKent, UK

Over the last 30 years, there has been increasing policy emphasis on care in community-based settings for those with dependency needs. This, coupled with the long-term shift towards an ageing population and the improved longevity of those with lifelong disabilities, has had significant implications for family carers. In the United Kingdom, there are currently 6.5 million carers; this figure is predicted to rise by 3.4 million by 2045 (Larkin & Milne 2013, Carers UK 2014). The intensity of care needs will also increase and more carers are likely to be supporting two people, e.g. a disabled partner *and* a parent. There is an expectation that carers will not only take responsibility for managing a greater range of, and often more complex, health conditions but that they will also have the capacity to do so. The predicted shortfall between the ‘demand’ for and ‘supply’ of carers will be reached in 2017 leading to what many predict will be a ‘crisis in care’ (Pickard 2013). Such concerns are amplified in an era of public sector austerity measures and the effective implementation of the Care Act (2014).

The national and international evidence base around family care has been growing since the 1980s. It can be credited, in part, for raising carers' profile in the public domain (Larkin & Milne 2013), ensuring the prioritisation of caring as a significant issue within social policy and practice and informing improvements in practice. However, despite the fact that much has been written about caring, there has been limited consideration of the nature of the evidence base and how it can contribute to improving carers' quality of life and those they support.

During a seminal Economic and Social Research Council ‘Carers Seminar Series’ (2012/13), the existing carer-related body of knowledge was reviewed. The overall aim of the series was to explore ways of maximising the utility and impact of research and develop a coherent evidence base about policy, services and interventions relating to carers. The seminars drew together key stakeholders in the carers field including academic researchers, early career researchers, carers, service users, third sector organisations, practitioners, government department representatives and postgraduate students. Key objectives included:

- reviewing the existing research evidence about the cost-effectiveness of policy and health and social care interventions for carers and those they support;
- establishing a future agenda for research around services that meet the needs of carers and improves both their quality of life and that of the relatives they support; and
- exploring key theoretical, conceptual and methodological challenges facing the research community.

A number of papers in this Special Issue of *Health and Social Care in the Community* are drawn from the ‘Carers Seminar Series’. The first paper by **Milne and Larkin** is intended to prompt debate about the nature of carer-related research and the powerful link between the type of research conducted and the generation of understanding and knowledge about care, carers and care-giving. Their critical analysis suggests that the majority of existing research can be located inside two distinctive and very separate research paradigms – ‘Gathering and Evaluating’ and ‘Conceptualising and Theorising’. The former provides evidence about the extent of care-giving; who provides care to whom and with what impact; and focuses on evaluating policy and service efficacy. The authors argue that this type of research tends to dominate public perception about caring and strongly influences the type and extent of policy and support for carers. In contrast, the latter explores the conceptual and experiential nature of care and aims to extend thinking and theory about caring. It is concerned with promoting understanding of care as an integral part of human relationships, embedded in the life course, and a product of interdependence and reciprocity. Milne and Larkin conclude that much could be gained for citizens, carers and families, and the generation of knowledge advanced, if the two bodies of research were integrated to a greater degree.

The papers by **Robinson and Seddon** and **Mitchell**, **Brooks and Glendinning** address carer assessment. They both explore the extent to which policies around carer assessment aimed at increasing carers' rights and enhancing their choice and control are operationalised in front-line practice. **Robinson and Seddon's** paper is based on a longitudinal study spanning over 20 years (1993–2013) and includes qualitative data from in-depth interviews with social care practitioners across England and Wales. Their study highlights significant and persistent tensions around the delivery and management of carer assessments, including practitioner ambivalence about the value of separate carer assessments, assumptions about carers' willingness and ability to continue to provide care, and failure to capture the reciprocal or mutual basis of much family care-giving**. Mitchell, Brooks and Glendinning** used online surveys, interviews and focus groups to explore carers' roles in relationship to assessment, support planning and personal budget (PB) allocation for older and people with a disability. They found that while carers played important roles in service users' assessments and support planning, they were less likely to report receiving assessments or support in their own right over which they can exercise choice and/or control. This study concludes that one of the underlying barriers to implementing policy aims around promoting carers' recognition and rights is the – often opaque – way that practitioners (continue to) conceptualise carers primarily as a resource, rather than as a recipient of care and support. Both studies draw attention to the challenges inherent in achieving personalised support for carers through the carers' assessment process and raise the question about the likely – negative – impact of further austerity measures on services for carers.

The focus of **Larkin's** paper is of linked relevance. Using semi-structured in-depth interviews, **Larkin** explored carers' perceptions of a change in the service user's support from local authority provided services to a PB on the carer–service user relationship. The study confirmed existing evidence that PBs can improve relationships between carers and service users, for example by accessing support which enables both parties to undertake activities individually and together. It also confirmed that PBs can have positive outcomes on carers' sense of control over their daily lives, quality of life, health and well-being. However, this study did show that in order for these outcomes to be achieved, carers need to have confidence in the quality of the care they can access. Some carers also reported needing more support and training with ‘paperwork and the recruitment and management of staff’ as they found these tasks particularly problematic. In addition, concerns about the impact of continuing budget cuts were identified as a source of stress for many carers.

**Moriarty's** paper presents findings from a study about how carers access information and assistance to which they are entitled. This topic has particular relevance given the emphasis on better help and advice for carers in the Care Act (2014). **Moriarty** used concurrent mixed methodology comprising semi-structured interviews and an email survey and identified a number of different models of outreach. These included self-help outreach, specialist outreach and outreach that is dependent on practitioners' ability to identify carers and explain to them what help is available. The findings suggest that carers' diverse situations and needs require there to be a range of different models of outreach service. Further research is needed to identify which particular models are most effective in terms of preventing or delaying carers' need for more intensive support.

The issue of carers' uptake of services is a key theme in two of the other papers in this Special Issue – those by **Neville** and **Greenwood**. Respectively, the papers are about dementia carers and ethnic minority carers, both of whom are growing in number in the United Kingdom. These two authors highlight the international evidence that, despite the positive impact of support from services on carer well-being, uptake remains low among these two groups of carers. The literature review conducted by **Neville** focuses on the limited use of respite care by dementia carers. While the review does not produce definitive conclusions, it identifies a number of barriers to uptake. These include failure to recognise the ‘need’ for respite, carers not allowing themselves ‘permission’ to utilise services (including respite care), limited service availability and concerns about quality. The author makes a number of suggestions about future research, such as gaining a greater understanding of the role played by respite in carers' lives and outcomes achieved; this question would best be answered using an in-depth qualitative approach. The relevance of such research for policy decisions about funding models and availability of respite services is also highlighted. **Greenwood** conducted a systematic review which aimed to explore minority ethnic carers' perceptions of barriers to service use. She concludes that while some barriers are specific to minority ethnic carers (such as cultural and religious appropriateness), many of the barriers identified are relevant to *all* carers. Examples include limited awareness of services, having insufficient information, a sense of duty, concerns over finances and reluctance on behalf of the care recipient to accept external help.

There are some interesting parallels between **Greenwood**'s paper and **Williams and Donovan's** paper about Vietnamese family caregivers domiciled in Ontario (Canada) providing end of life care. This longitudinal study used an instrumental case study methodology designed to capture changes experienced by carers in the care-giving situation over time. The authors conclude that in order for this group of carers to be effectively supported, services need to be underpinned and developed by a better understanding of the cultural context and needs of Vietnamese families. Services need to be more culturally and linguistically appropriate, family oriented (rather than patient centred), respectful of privacy and modesty, and tolerant of multiple healthcare approaches (i.e. both western and traditional medicine). Food preferences were also identified as important.

One of the consequences of the increase in the number of carers, especially older carers, is that there are more people who are ‘former carers’, i.e. carers whose relative has died. Indeed every year, 2 million people are in this situation (Carers UK 2013). Despite acknowledgement of this fact, life post caring is still relatively underexplored. **Hynes** ***et al.*****'s** study in Ireland builds on previous research about the impact of caring ceasing on carers' lives (Larkin 2009). Their findings identified a number of threats to former carers' health and well-being; financial problems and social isolation are particular issues. The authors conceptualised the post-caring trajectory as a time of being ‘between worlds’ comprising three iterative interrelated transitions: ‘loss of the caring world’, ‘living in loss’ and ‘moving on’. They also highlight the need for further research into the ‘legacies of caring’ and the need for targeted support that is sensitive to the different experiences of former carers and to the specific phases of the post-caring experience.

The collection of papers in this Special Issue reflects a number of key dimensions of carer-related research more widely. Carer-related research is characterised by a range of different methodologies and focuses on different groups of carers caring in a range of contexts. Research relating to the impact and efficacy of policy and to the translation of policy aims into front-line practice is also a long-standing feature of research in the carers' field. A commitment to capturing carers' experiences and lives and recognition of the very different needs and profiles of carers is also an enduring dimension. In addition, this Special Issue includes papers presenting original research from Ireland and Canada and literature reviews that draw on international evidence; this is indicative of the increasing visibility of care-related issues on the international stage.

The papers also highlight a number of the tensions that underpin research on carers and caring. Responses to addressing ‘barriers’ to help seeking and service uptake often require investment of public resources. At a time when more is expected of carers and less is being provided by health and social care services, this may be unrealistic. It is important to recognise that carers' assessments are being conducted in a context where local authorities in England are raising their eligibility thresholds and reducing access to services. How far a practitioner can offer help in an environment of severe cost constraint is therefore a significant challenge. A third issue relates to who is the focus of research. Most research on carers is done with those who define themselves as carers. What we do know is that as many as half of those who actually ‘do care-giving’ do not view themselves as a carer and as such, tend to be marginal to the purview of research (Lloyd 2006). The groups most ‘in need’ of recognition and support are often those least likely to come forward for help or seek out a ‘carer’-related service. A single collection of papers can only ever ‘represent’ a small part of the multidimensional, heterogeneous, diverse and shifting population that constitute carers. However, it does showcase the potential of research to capture carers' experiences and lives, to expose the impact of cuts to services and the challenges of implementing policy change, explore the role of assessment and support services, and challenge the research community to review how it generates knowledge and develops understanding about carers, care and caring.
